# Case of Plica Polonica

**Published:** 1806-09

**Authors:** M. Defour

**Affiliations:** Physician to the Imperial Hospital of Quinze-Vingts, at Paris


### Case of Plica Polonica, by M. Defour, *Physician to the Imperial Hospital of Quinze-Vingts, at Paris.*

In this disease the hairs of the head are so twisted and interlaced with each other, that they cannot be separated without tearing or breaking them asunder, in which case they pour out a bloody fluid. The plica polonica is almost unknown in France, but is so extremely prevalent in Poland, as to have obtained the above appellation. The Jews residing in that country are particularly subject to it. Among the causes principally operating to produce this cutaneous affection, is reckoned the slovenly and dirty manner in which these people live; they seldom comb their hair; they live in low and moist situations, and indulge to excess in the use of spirituous liquors. Another cause believed to be productive of this malady is the use of certain waters, which, whether employed in the way of drink, or under the form of baths, never fail to produce disagreeable consequences. To these causes must be superadded, an hereditary taint transmitted from father to son, and which consists in the too great relaxation of the pores and bulbs of the hairs, which are situated beneath the skin of the cranium. Thus it happens that a gross juice or matter, generated by the use of coarse or indigestible aliments, and of impure water, is propelled, by means of the heat excited in consequence of the abuse of spirituous liquors, into the cavities of the hairs, oozes through their pores, and occasions this malady.

During the month of December, 1801, I was desired to visit Mad. C—, residing in Vendome-street, who laboured under this malady. This lady was of a sanguine temperament, and of an irritable disposition. During her youth, she had suffered much from violent affections of the mind, and had been much addicted to the use of spirituous liquors. I saw her a few days after the disease manifested itself, and found her labouring under an acute inflammatory fever; her eyes appeared extremely red, remained half shut, and could scarcely tolerate the faintest light. On the fifteenth day from the commencement of the disease, I observed that the hairs stood erect, and were so intertwined, and glued together, that a night cap could not be kept on her head; and when the matted hairs were either cut, or broken, they poured out blood, especially during the paroxysms, or exacerbations of the fever, because, cause, at these periods, the patient was much more agitated, and more frequently moved her head.

During the first and second stages of the disease, I prescribed cooling, diluting, and demulcent medicines; and, in the third stadium, bitters combined with acids, and occasionally gentle aperients were administered. At the same time, I employed externally, as a lotion, a decoction of comfrey roots, the leaves of brank-ursine, marsh-mallows, groundsel, and other emollients, with a view to favour the expulsion of the morbific matter by the hair, which appeared to be the outlet pointed out by Nature, as well as to prevent the reabsorption of the acrimonious and putrid humour into the system, and thus prevent its repulsion upon any of the internal viscera.

After a space of three months, during which period much blood and pus were discharged, the complaint at last yielded without cutting out the hair, a practice resorted to in Poland; and on the eighty-third day of the disease, to the astonishment of her friends, and physician, the hairy scalp entirely separated, and was found lying loose within her night-cap.

The patient long preserved this integument of the cranium, which was extremely curious, not only on account of the size, or dilatation of the hairs, but from their matted form.

Since her recovery, a new crop of hair has made its appearance, and at the present period, 1806, is nearly as luxuriant as formerly.

